# Transforming and extending library services by embracing technology and collaborations: A case study

**DOI:** 10.1111/hir.12439

**Published:** 2022-06-22

**Authors:** Terrie R. Wheeler, Diana Delgado, Paul J. Albert, Sarah Ben Maamar, Peter R. Oxley

**Affiliations:** ^1^ Weill Cornell Medicine Samuel J. Wood Library and C.V. Starr Biomedical Information Center New York New York USA; ^2^ Information, Education and Clinical Services Weill Cornell Medicine Samuel J. Wood Library and C.V. Starr Biomedical Information Center New York New York USA; ^3^ Information Technologies & Services Weill Cornell Medicine New York New York USA

**Keywords:** artificial intelligence (AI), bibliometrics, clinical decision making, consumer health information, information services, libraries, academic, libraries, medical, library services, research data (management), research support

## Abstract

Technology advances and collaborations with information technology and computer science groups have enabled library services to expand into new domains. Listening to user needs, eliminating administrative burden and saving users time remain strong foundations on which to build new library services enabled by technology. Examples of what is now possible is described, including service to user groups, successes, failures and challenges. Although technology advances have enabled library service enhancements to all user groups, special emphasis on new library services in support of the research enterprise is discussed. As Lindberg and Humphreys predicted in 2015, the research enterprise's need for responsible curation of research data has created new opportunities for library services and examples of those services are discussed. As technology continues to advance, new library services are expected to emerge. These may include regulatory and compliance services. By developing these services with user feedback to save users time and expedite their work, and in collaboration with technology experts, libraries can expect to offer sustainable and valued services for years to come.

## INTRODUCTION

In her editorial introducing this series, Jeannette Murphy described how Health Sciences Libraries (HSLs) increasingly embraced technology. She noted the accuracy of Lindberg and Humphrey's predictions for 2015 (Lindberg & Humphreys, 2005), a frame used here to describe the technological advances of HSLs in North America, and specifically the Samuel J. Wood Library at Weill Cornell Medicine. HSLs today serve as a technology hub (Patterson et al., 2018) by identifying technology trends to improve service provision (Norton et al., 2018), and teaching users to optimise technology (Hurst, 2014). Lindberg and Humphreys looked at HSL services to users broadly, as well as to specific groups such as clinicians, consumers, medical students, and researchers. The Wood library collections are delivered to users broadly via the library's web page, and browser extensions enable user interaction with licensed content on any website the user is browsing. The library's webpage supports chat reference and forms for online, interactive reference. Wood Library technology services arose through user demand or institutional need.

## HOW THE WOOD LIBRARY'S APPROACHES SERVICE DEVELOPMENT

The Wood Library's philosophy in developing a service is to reduce either time spent or the administrative burden on the user. Because the services described below enabled the researcher to spend more time on science and the clinician to spend more time seeing patients, these innovations have been well received, which is key to their success and adoption by the user community.

## USING TECHNOLOGY TO ENHANCE LIBRARY SERVICES FOR USER GROUPS

### Clinicians

As Lindberg and Humphreys envisioned, HSL services are delivered to clinicians ‘in context’ (Lindberg & Humphreys, 2005) by librarians with subject expertise at morning report, rounds, and other venues using electronic devices. Morning report is a departmental meeting for residents led by attending physicians, which provides a forum for residents to discuss daily cases they see in the hospital and offer their differential diagnosis and treatment plan based on history and physical, chief complaint, current drug regimen, and the results of diagnostic tests ordered. Rounds is a similar process done at the patient's bedside. Prior to the COVID‐19 pandemic rounds and morning report were primarily in person with various types of reference services being delivered electronically through e‐mail, Microsoft Teams, web pages, libguides, chat service, or by consulting with a librarian. The Wood Library delivers HSL services such as decision support electronically via a webpage or libguide, an app on a mobile device, or via the electronic health record (EHR). With the advent of the pandemic, morning report moved to Zoom, and rounds ceased. Decision support consists of taking a specific patient's medical information (chief complaint, drug regimen, test results) and comparing it with the literature in real time through decision support tools embedded in the EHR that aid clinicians in making the best clinical decisions possible with available evidence.

### Consumers

Another HSL service that WCM offers to in‐patients, outpatients and community members is consumer health information (CHI) (DeRosa et al., 2019). This is offered either in person via the Patient Resource Center, via rounds or via a consult through the EHR. Access to the EHR is critical for librarians to efficiently deliver CHI to specific patients upon a referral from another health care team member. The librarian is a recognised member of the health care team whose role is to identify evidence based information and who is bound by the same privacy requirements as other team members. Another CHI service is group educational sessions on various health topics. Prior to the pandemic these were offered in person but have become more popular and easier to consume when offered virtually over Zoom. CHI is delivered in person in the Patient Resource Center and on rounds, electronically via website or e‐mail, or less often over the phone.

### Medical students

Library instruction in medical education across North America is offered both synchronously and asynchronously, covering an increasingly wider range of topics (Nevius et al., 2018). At WCM librarians serve as instructors or lecturers of evidence‐based practice where students learn to appraise the medical literature and develop a diagnosis and treatment plan for patients. Librarians also support faculty in creating online courses. WCM student‐centered technology systems include Canvas, the student learning management system, various audience response systems to keep students engaged in learning, and Panopto for educational videos. The physical library is equipped with Mac and PC workstations throughout and serves as an incubator where students can use a variety of technologies to support their learning 24 hours a day.

### Researchers

Lindberg and Humphries imagined digital libraries would feature rich interconnections between various types of research data, aggregated clinical data, and published literature (Lindberg & Humphreys, 2005) to serve the research enterprise. WCM has leveraged technology to offer robust HSL services for researchers such as systematic reviews, publication reporting, bioinformatics, scientific software, and data management services.

## USING TECHNOLOGY TO DEVELOP LIBRARY RESEARCH SUPPORT SERVICES

### Systematic reviews

Many North American libraries use specialised software (Harrison et al., 2020) to efficiently manage the systematic review process. The Wood Library uses Covidence, a systematic review software suite. Librarians are involved in multiple stages of the systematic review process, including formulating the review question, protocol development, comprehensive searches, reference chasing, and writing the review. Library support staff upload full text and request interlibrary loans for selected papers. For a sense of volume, in 2021 the Wood Library received 60 systematic review requests and published 30.

### Publication reporting

The Wood Library is responsible for WCM's faculty profiles, and uses VIVO open source technology (Dutta, 2022c) to host these profiles. In a related project, the Wood Library has developed a publications management system including a reporting component called ReCiter (Albert et al., 2021) which uses machine learning to identify publications written by WCM researchers. ReCiter (Dutta, 2022b) is open‐source code available for any library to download. The Wood Library also has a one‐click bibliometric report generation tool (Dutta, 2022a) which can produce a document describing any WCM researcher's publication impact, benchmarking the researcher against peers using NIH's iCite service (Hutchins et al., 2016). Network visualisations can be created from this publication report data using a tool like vis.js (VIS.JS, n.d.).

### Bioinformatics service and scientific software hub

The Wood Library's Bioinformatics Service (Wheeler & Oxley, 2018) targets investigators who need basic support with analysing their bioinformatics data. Some Bioinformatics Service collaborations have produced high‐impact publications (Rodrigues et al., 2019). This service provides data analysis for researchers and consultations on analysis software available via the Library Scientific Software Hub. The Library's Scientific Software Hub (Weill Cornell Medicine, 2022b) is an online catalogue of all WCM licensed scientific software describing system requirements and links to tutorials. The Hub provides download links to open access software, or else a request form is presented to allow the library to provision licensed software.

### Data management services

Listening to researcher needs helps HSLs develop future services. In 2016 WCM researchers described a Data Core to library leadership. A Data Core is a protected environment which allows researchers to access both their confidential data and computational resources to analyse massive data sets such as Medicare data, the INSIGHT Clinical Research Network Patient Centered Outcomes Research Institute (PCORI), or the COVID‐19 Data Lake, all studies with WCM investigators. Through partnerships with WCM research departments and WCM Information Technology and Services (ITS), today the library manages this Data Core (Oxley et al., 2018) and has more than 150 projects with nearly 400 active users. To make findable and as accessible as permitted by law, the data in the Data Core (Weill Cornell Medicine, 2022c) is now displayed in a Data Catalogue (Weill Cornell Medicine, 2022a). The library is currently extending the data catalogue with archival capacity and a ‘data retention wizard’ to allow researchers to submit biological data sets and their metadata associated with publications and grants, so that researchers will be able to comply with the National Institutes of Health (NIH) *Final NIH Policy for Data Management and Sharing* (NIH, 2020), effective January 2023. This policy will position HSL's in the United States to offer data management services to researchers and clinicians working with large data sets. Lindberg and Humphreys foresaw the library's critical role to ‘store and manage access to electronic scientific and health‐related data’ curating what they called ‘the flood of patient‐specific data’ (Lindberg & Humphreys, 2005).

### Nascent research services

Research reproducibility has become increasingly important as more medical research studies are published that are either not reproducible, or whose findings are challenged. WCM's Research Integrity Office has asked the Wood Library to develop an image forensics service that would enable Western blot images to be evaluated with a deep‐learning approach that would enable the bioinformatics librarian to quickly identify images that have been manipulated. In collaboration with Cornell Tech and Elisabeth Bik (Bik et al., 2016), the Wood library is building a repository for western blot and other molecular biology images to improve the accuracy of these machine learning models. The Cornell Tech students host this image library in the cloud, and it is accessible via a non‐public website behind our firewall. This nascent service is an example of how librarians collaborate with graduate level computer science students to exploit technology to deliver new and needed services to their organisation.

Lindberg and Humphreys further envisioned the typical Wood librarian by stating ‘many librarians have advanced training in both subject matter disciplines and information science. It is common to find librarians working as part of health care teams, writing grant proposals, serving on institutional review boards, … working as faculty members in evidence‐based medicine courses’. (Lindberg & Humphreys, 2005).

## CHALLENGES FACING HEALTH SCIENCE LIBRARIES

When introducing new technologies, the biggest challenge is integrating intuitive tools into the user workflow so that users do not have to spend time learning to use the service. Therefore, user feedback in development is critical. Another challenge is greater demand for service than capacity, which requires that librarians work in teams and maintain policies regarding service access to ensure equity across user groups. Another challenge is the expectation that all library services are free. In some cases, such as the Data Core, advanced technologies (server, software, storage costs) require cost recovery set by the institution to keep the service operational.

## FAILURES

WCM is a learning organisation, meaning that we tolerate failures and embrace the lessons learned from them. One such failure is a custom home‐grown application called VIVO Dashboard. VIVO Dashboard offered users the ability to interact with various bibliometric statistics which speak to the research impact of each researcher, each department, and of the entire medical school. Although we invested many resources into developing VIVO Dashboard at the request of WCM's Research Dean, it had several key shortcomings. It was not architected to nimbly accommodate the sheer volume of publication metadata loaded into it, and the need to update data daily. Also, we anticipated that the system would be widely used by WCM administrators, but it was not. Even though VIVO Dashboard offered an interactive interface that allows users to serendipitously explore the publication output of a given researcher, what its users really wanted was a single, quantitative metric, which speaks to a researcher's impact in comparison to peers. Although h‐index has its shortcomings, unfairly benefitting those with long careers, it is one such metric. We have attempted to address these problems with our new one‐click bibliometric report generation tool described above under publication reporting.

## CONCLUSION

Most new services developed by the Wood Library were suggested by WCM administrators to reduce administrative burden and meet user needs. Following Ranganathan's fourth law of Library Science, these innovations ‘saved the time of the reader’ (Ranganathan, 1931). Some new services also help with regulatory compliance and encourage research reproducibility. Wood librarians have responded well to requests for new services by responsibly developing them with user feedback, and in collaboration with technology experts in ITS and Cornell Tech. If librarians listen to the needs of their users and deliver responsibly developed services, the HSL's future of embracing technology is bright.

## CONFLICT OF INTEREST

None.

## References

[hir12439-bib-0001] Albert, P. J. , Dutta, S. , Lin, J. , Zhu, Z. , Bales, M. , Johnson, S. B. , Mansour, M. , Wright, D. , Wheeler, T. R. , & Cole, C. L. (2021). ReCiter: An open source, identity‐driven, authorship prediction algorithm optimized for academic institutions. PLoS ONE, 16(4), e0244641. 10.1371/journal.pone.0244641 33793563 PMC8016248

[hir12439-bib-0002] Bik, E. M. , Casadevall, A. , Fang, F. C. , & Sibley, L. D. (2016). The prevalence of inappropriate image duplication in biomedical research publications. mBio, 7(3), e00809–e00816. 10.1128/mBio.00809-16 27273827 PMC4941872

[hir12439-bib-0003] DeRosa, A. P. , Baltich Nelson, B. , Delgado, D. , Mages, K. C. , Martin, L. , & Stribling, J. C. (2019). Involvement of information professionals in patient‐ and family‐centered care initiatives: A scoping review. Journal of the Medical Library Association, 107(3), 314–322. 10.5195/jmla.2019.652 31258437 PMC6579588

[hir12439-bib-0004] Dutta, S . (2022a). ReCiter machine learning/analysis—A suite of scripts and tools for retrieving and analyzing data from ReCiter. https://github.com/wcmc-its/reciter-machineLearning-Analysis/

[hir12439-bib-0005] Dutta, S . (2022b). ReCiter: An enterprise open source author disambiguation system for academic institutions. https://github.com/wcmc-its/ReCiter

[hir12439-bib-0006] Dutta, S . (2022c). WCMC‐ITS/VIVO‐Docker. https://github.com/wcmc-its/vivo-docker

[hir12439-bib-0007] Harrison, H. , Griffin, S. J. , Kuhn, I. , & Usher‐Smith, J. A. (2020). Software tools to support title and abstract screening for systematic reviews in healthcare: An evaluation. BMC Medical Research Methodology, 20(1), 7. 10.1186/s12874-020-0897-3 31931747 PMC6958795

[hir12439-bib-0008] Hurst, E. J. (2014). Educational technologies in health sciences libraries: Teaching technology skills. Medical Reference Services Quarterly, 33(1), 102–108. 10.1080/02763869.2013.866494 24528269 PMC3943340

[hir12439-bib-0009] Hutchins, B. I. , Yuan, X. , Anderson, J. M. , & Santangelo, G. M. (2016). Relative citation ratio (RCR): A new metric that uses citation rates to measure influence at the article level. PLoS Biology, 14(9), e1002541. 10.1371/journal.pbio.1002541 27599104 PMC5012559

[hir12439-bib-0010] Lindberg, D. A. , & Humphreys, B. L. (2005). 2015—The future of medical libraries. The New England Journal of Medicine, 352(11), 1067–1070. 10.1056/NEJMp048190 15784661

[hir12439-bib-0011] Nevius, A. M. , Ettien, A. , Link, A. P. , & Sobel, L. Y. (2018). Library instruction in medical education: A survey of current practices in the United States and Canada. Journal of the Medical Library Association, 106(1), 98–107. 10.5195/jmla.2018.374 29339939 PMC5764599

[hir12439-bib-0012] NIH . (2020). Final NIH policy for data management and sharing. https://grants.nih.gov/grants/guide/notice-files/NOT-OD-21-013.html

[hir12439-bib-0013] Norton, H. F. , Tennant, M. R. , Edwards, M. E. , & Pomputius, A. (2018). Use of annual surveying to identify technology trends and improve service provision. Journal of the Medical Library Association, 106(3), 320–329. 10.5195/jmla.2018.324 29962910 PMC6013140

[hir12439-bib-0014] Oxley, P. R. , Ruffing, J. , Campion Jr, T. R. , Wheeler, T. R. , & Cole, C. L. (2018). Design and implementation of a secure computing environment for analysis of sensitive data at an academic medical center. Paper presented at the AMIA Annual Symposium Proceedings. PMC6371349 30815128

[hir12439-bib-0015] Patterson, B. , Casucci, T. , Hull, B. E. , & Lombardo, N. T. (2018). Library as the technology hub for the health sciences. Medical Reference Services Quarterly, 37(4), 341–356. 10.1080/02763869.2018.1514899 30722771

[hir12439-bib-0016] Ranganathan, S. R. (1931). The five laws of library science. Madras Library Association.

[hir12439-bib-0017] Rodrigues, G. , Hoshino, A. , Kenific, C. M. , Matei, I. R. , Steiner, L. , Freitas, D. , Kim, H. S. , Oxley, P. R. , Scandariato, I. , Casanova‐Salas, I. , Dai, J. , Badwe, C. R. , Gril, B. , Tešić Mark, M. , Dill, B. D. , Molina, H. , Zhang, H. , Benito‐Martin, A. , Bojmar, L. , … Lyden, D. (2019). Tumour exosomal CEMIP protein promotes cancer cell colonization in brain metastasis. Nature Cell Biology, 21(11), 1403–1412. 10.1038/s41556-019-0404-4 31685984 PMC7354005

[hir12439-bib-0018] VIS.JS . (n.d.). Network. https://visjs.github.io/vis-network/docs/network/

[hir12439-bib-0019] Weill Cornell Medicine . (2022a). Data catalog. https://its.weill.cornell.edu/services/research-informatics/data-catalog

[hir12439-bib-0020] Weill Cornell Medicine . (2022b). Library scientific software hub. https://library.weill.cornell.edu/research-support/library-scientific-software-hub

[hir12439-bib-0021] Weill Cornell Medicine . (2022c). WCM data core. https://its.weill.cornell.edu/services/research-informatics/wcm-data-core

[hir12439-bib-0022] Wheeler, T. R. , & Oxley, P. R. (2018). Developing a library bioinformatics program fully integrated into a medical research institution. Medical Reference Services Quarterly, 37(4), 413–421. 10.1080/02763869.2018.1514915 30722773

